# Supplementary material for: Novel Type of Tetranitrosyl Iron Salt: Synthesis, Structure and Antibacterial Activity of Complex [FeL’2(NO)2][FeL’L”(NO)2] with L’-thiobenzamide and L”-thiosulfate
Source: Molecules. 2022 Oct 14;27(20):6886. doi: 10.3390/molecules27206886 (PMC9611265; doi:10.3390/molecules27206886)

checkCIF/PLATON report

Structure factors have been supplied for datablock(s) Compound_I

THIS REPORT IS FOR GUIDANCE ONLY. IF USED AS PART OF A REVIEW PROCEDURE FOR PUBLICATION, IT SHOULD NOT REPLACE THE EXPERTISE OF AN EXPERIENCED CRYSTALLOGRAPHIC REFEREE.

No syntax errors found. CIF dictionary Interpreting this report

Datablock: Compound_I

```
Bond precision:   C-C = 0.0044 A          Wavelength=0.78790

Cell:               a=11.047(2)      b=23.433(5)        c=11.611(2)
                    alpha=90         beta=95.10(3)      gamma=90
Temperature:        100 K
```

	Calculated	Reported
Volume	2993.8(10)	2993.8(10)
Space group	P 21/c	P 21/c
Hall group	-P 2ybc	-P 2ybc
Moiety formula	C14 H14 Fe N4 O2 S2, C7 H7 Fe N3 O5 S3	C14 H14 Fe N4 O2 S2, C7 H7 Fe N3 O5 S3
Sum formula	C21 H21 Fe2 N7 O7 S5	C21 H21 Fe2 N7 O7 S5
Mr	755.45	755.45
Dx,g cm-3	1.676	1.676
Z	4	4
Mu (mm-1)	1.811	1.811
F000	1536.0	1536.0
F000'	1542.47	
h,k,lmax	14,30,15	14,30,15
Nref	7010	6744
Tmin,Tmax	0.805,0.834	0.770,0.810
Tmin'	0.762	

```
Correction method= # Reported T Limits: Tmin=0.770 Tmax=0.810
AbsCorr = MULTI-SCAN

Data completeness= 0.962           Theta(max)= 31.018

R(reflections)= 0.0388( 5392)          wR2(reflections)= 0.1012( 6744)

S = 1.022                 Npar= 398
```

The following ALERTS were generated. Each ALERT has the format
test-name_ALERT_alert-type_alert-level.
Click on the hyperlinks for more details of the test.

Alert level B

```
PLAT430_ALERT_2_B Short Inter D...A Contact  O1       ..O3       .       2.79 Ang.
                                            1+x,y,z  =      1_655 Check
```

Alert level C

```
PLAT911_ALERT_3_C Missing FCF Refl Between Thmin & STh/L=    0.600        244 Report
```

Alert level G

```
ABSMU01_ALERT_1_G  Calculation of _exptl_absorpt_correction_mu
                not performed for this radiation type.
PLAT019_ALERT_1_G _diffrn_measured_fraction_theta_full/*_max < 1.0      0.993 Report
PLAT092_ALERT_4_G Check: Wavelength Given is not Cu,Ga,Mo,Ag,In Ka    0.78790 Ang.
PLAT232_ALERT_2_G Hirshfeld Test Diff (M-X)  Fe1      --N2       .        6.6 s.u.
PLAT232_ALERT_2_G Hirshfeld Test Diff (M-X)  Fe2      --N5       .        7.2 s.u.
PLAT232_ALERT_2_G Hirshfeld Test Diff (M-X)  Fe2      --N6       .        5.1 s.u.
PLAT910_ALERT_3_G Missing # of FCF Reflection(s) Below Theta(Min).          1 Note
PLAT912_ALERT_4_G Missing # of FCF Reflections Above STh/L=  0.600         22 Note
PLAT913_ALERT_3_G Missing # of Very Strong Reflections in FCF ....          1 Note
PLAT941_ALERT_3_G Average HKL Measurement Multiplicity ..........         2.2 Low
PLAT978_ALERT_2_G Number C-C Bonds with Positive Residual Density.          3 Info
```

0 **ALERT level A** = Most likely a serious problem - resolve or explain
1 **ALERT level B** = A potentially serious problem, consider carefully
1 **ALERT level C** = Check. Ensure it is not caused by an omission or oversight
11 **ALERT level G** = General information/check it is not something unexpected

2 ALERT type 1 CIF construction/syntax error, inconsistent or missing data
5 ALERT type 2 Indicator that the structure model may be wrong or deficient
4 ALERT type 3 Indicator that the structure quality may be low
2 ALERT type 4 Improvement, methodology, query or suggestion
0 ALERT type 5 Informative message, check

It is advisable to attempt to resolve as many as possible of the alerts in all categories. Often the minor alerts point to easily fixed oversights, errors and omissions in your CIF or refinement strategy, so attention to these fine details can be worthwhile. In order to resolve some of the more serious problems it may be necessary to carry out additional measurements or structure refinements. However, the purpose of your study may justify the reported deviations and the more serious of these should normally be commented upon in the discussion or experimental section of a paper or in the "special_details" fields of the CIF. checkCIF was carefully designed to identify outliers and unusual parameters, but every test has its limitations and alerts that are not important in a particular case may appear. Conversely, the absence of alerts does not guarantee there are no aspects of the results needing attention. It is up to the individual to critically assess their own results and, if necessary, seek expert advice.

Publication of your CIF in IUCr journals

A basic structural check has been run on your CIF. These basic checks will be run on all CIFs submitted for publication in IUCr journals (*Acta Crystallographica*, *Journal of Applied Crystallography*, *Journal of Synchrotron Radiation*); however, if you intend to submit to *Acta Crystallographica Section C* or *E* or *IUCrData*, you should make sure that full publication checks are run on the final version of your CIF prior to submission.

Publication of your CIF in other journals

Please refer to the *Notes for Authors* of the relevant journal for any special instructions relating to CIF submission.

PLATON version of 18/05/2022; check.def file version of 17/05/2022

Datablock Compound_I - ellipsoid plot

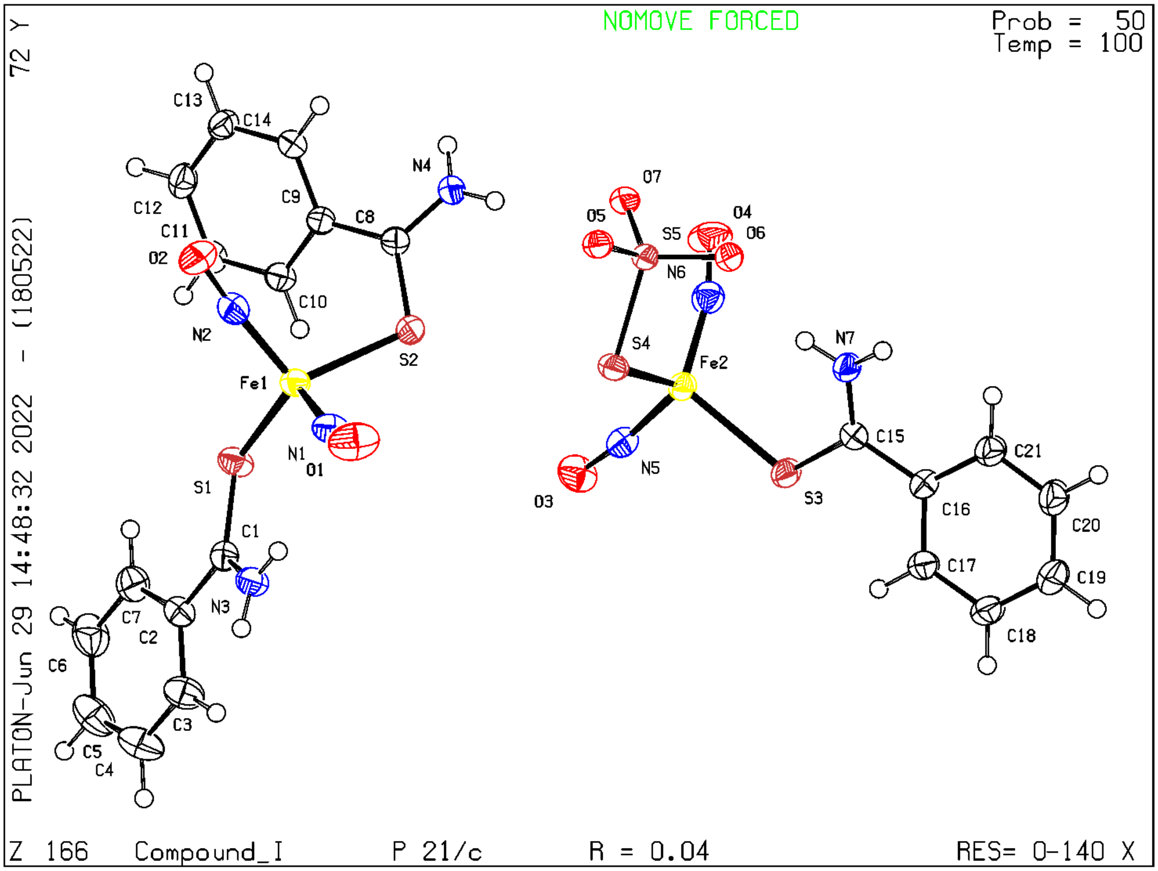

Supplement: Supplementary file 1 [file molecules-27-06886-s001.zip › checkcif_compound_I.pdf]
