# Supplementary material for: Novel Type of Tetranitrosyl Iron Salt: Synthesis, Structure and Antibacterial Activity of Complex [FeL’2(NO)2][FeL’L”(NO)2] with L’-thiobenzamide and L”-thiosulfate
Source: Molecules. 2022 Oct 14;27(20):6886. doi: 10.3390/molecules27206886 (PMC9611265; doi:10.3390/molecules27206886)
Supplement: Supplementary file 1 [file molecules-27-06886-s001.zip › Supplimentary Materials_red_13_10_2022.pdf]

“Supplementary Materials”

Novel type of tetranitrosyl iron salt: synthesis, structure and antibacterial activity of complex $[FeL'_2(NO)_2][FeL'L''(NO)_2]$ with L’- thiobenzamide and L”- thiosulfate

Nataliya A. Sanina*[a,b,c], Arina A. Starostina[b], Andrey N. Utenyshev[a], Pavel V. Dorovatovskii[d], Nina S. Emel’yanova[a,b], Vladimir B. Krapivin[a], Victor B. Luzhkov[a,b], Viktoriya A. Mumyatova[a], Anastasiya A. Balakina[a], Alexey A. Terent’ev[a,b,c], Sergey M. Aldoshin[a,b]

[a] *Federal Research Center of Problems of Chemical Physics and Medicinal Chemistry, RAS, 1 Acad. Semenov Av., 142432 Chernogolovka, Moscow Region, Russian Federation*

[b] *Faculty of Fundamental Physical-Chemical Engineering of M.V. Lomonosov MSU, Leninskie gory, 119991, Moscow, GSP-1, Russian Federation*

[c] *Scientific and Educational Center of the Moscow Regional State University in Chernogolovka Medical Biological Institute, 24, Vera Voloshina street, Mytischi, Moscow Region, 141014, Russia*

[d] *National Research Centre "Kurchatov Institute", 1 Acad. Kurchatov Square, 123182 Moscow, Russian Federation*

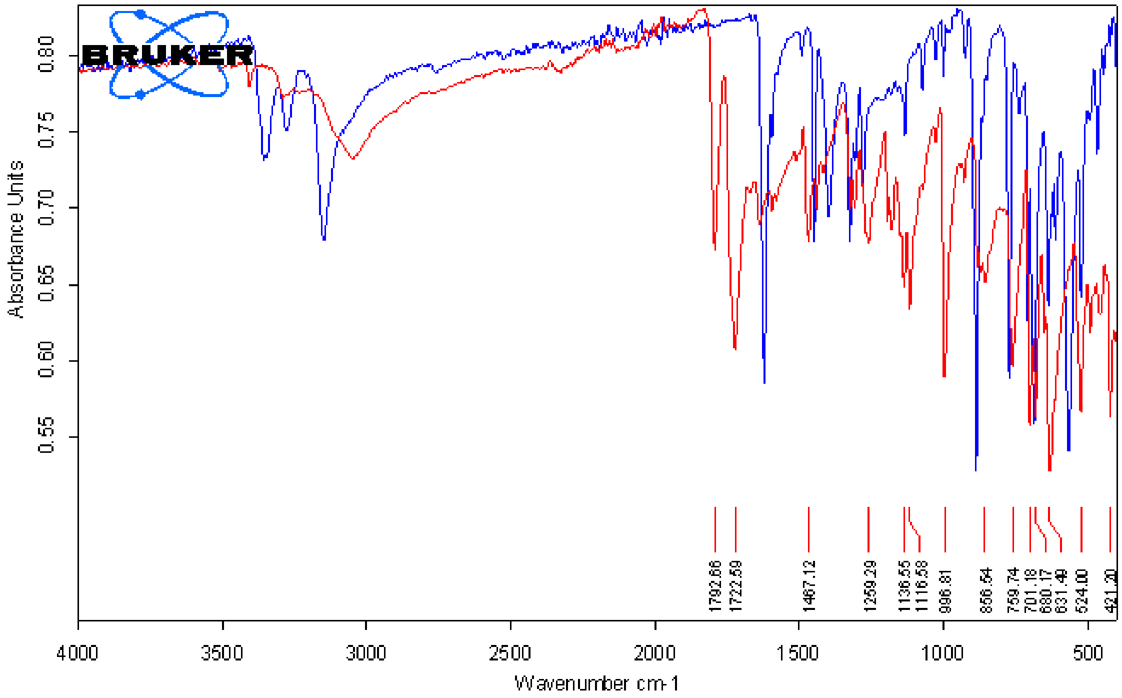

Fig.S1. IR spectra of **I** (red) and thiobenzamide (blue).

Table S1. Energies of symmetrical and most stable nonsymmetrical conformations of ***1***.

**Conformation**	$E_{el,w}$, a.u.	*ZPVE*, a.u.	$\Delta E_{0,w}$, kJ/mol
Symmetry C_2			
c1 (g+ g– g–)	-2971.30601024	0.268640	10.35
c2 (g+ tr g–)	-2971.31143513	0.270122	**0.00**
c3 (g+ g+ g+)	-2971.30545921	0.268135	10.47
c5 (g+ tr g+)	-2971.31113309	0.270268	1.18
c7 (g+ g– g+)	-2971.30131075	0.267708	20.24
c9 (tr g– g–)	-2971.30635972	0.268582	9.28
c12 (tr tr g+)	-2971.30894817	0.269852	5.82
Symmetry C_s			
c13 (g+ g+ g+)	-2971.30635827	0.268943	10.23
c15 (g+ tr g+)	-2971.31085030	0.270183	1.70
c16 (g+ tr g–)	-2971.31109733	0.270271	1.28
c19 (tr tr g+)	-2971.30915370	0.270980	8.24
Asymmetrical			
c20 (g+ tr g+/tr tr g–)	-2971.30994537	0.269369	1.93
c21 (g+ tr g–/tr tr g+)	-2971.30974319	0.269528	2.88
c22 (g+ tr g+/tr tr g+)	-2971.31034504	0.270069	2.72
c23 (g+ tr g–/tr tr g-)	-2971.31079689	0.270262	2.04
c24 (g+ tr g–/g+ g– g–) crystal. struct.	-2971.30869601	0.269637	5.92

[a] – *$E_{el,w}$ is the total electron energy in the water environment, ZPVE is the zero-point energy, $\Delta E_{0,w}$ is the relative total energy of the conformers*

Table S2. Energy of conformations of complex ***2***.

**Conformation**	$E_{el,w}$, a.u.	*ZPVE*, a.u.	$\Delta E_{0,w}$, kJ/mol
c1 (g+ tr g+) crystal. struct.	-3269.72277385	0.155024	1.24
c2 (g+ tr g–)	-3269.72338880	0.155167	**0.00**
c3 (g+ g+ g+)	-3269.71677075	0.154967	16.85
c6 (g+ g– g–)	-3269.71302400	0.154760	26.14
c7 (g– tr g–)	-3269.71941248	0.155251	10.66
c8 (g– tr g+)	-3269.72018780	0.154487	6.62
c9 (g– g– g–)	-3269.71591287	0.154659	18.29
c12 (g– g+ g+)	-3269.71616076	0.154842	18.12
c15 (g+ g– g–)	-3269.71483811	0.154796	21.48
c16 (tr tr g+)	-3269.71987325	0.155901	11.16
c17 (tr tr g–)	-3269.71983628	0.155592	10.44
c18 (tr g+ g+)	-3269.71683191	0.154756	16.14
c21 (tr g– g–)	-3269.71673288	0.154953	16.91

[a] – *$E_{el,w}$ is the total electron energy in the water environment, ZPVE is the zero-point energy, $\Delta E_{0,w}$ is the relative total energy of the conformers*

Conformational structures of complex **I** (complex *1*) optimized by the B3LYP/6-31+G(d,p) method.

Conformation c1


```
1\1\GINC-NODE159\FOpt\UB3LYP\6-31+G(d,p)\C14H14Fe1N4O2S2\SDS\11-Mar-20
21\0\\#N UB3LYP/6-31+G(d,p) Int(Grid=UltraFine) SCF(QC,Tight) SCRF=(Re
ad,Solvent=Water,SMD) Opt=(Redun) Freq(NoRaman) Symmetry(None) test\\[
Fe(NO)2(TBA)2]+ conf1 opt water\\1,2\Fe,0.0063153828,0.0024854085,1.00
02142199\S,1.8148720146,0.9165443394,-0.2934549937\S,-1.8032357432,-0.
9113829877,-0.2921952895\O,-1.2256669788,2.065473587,2.6479840046\O,1.
2375056654,-2.0640225942,2.6443629628\N,-0.7680741174,1.2959364639,1.8
822857068\N,0.7810222221,-1.2920034672,1.8805239074\N,2.6308059746,3.3
844637304,-0.6251793827\H,2.5440555848,4.3646892189,-0.8756461757\H,3.
5640399646,3.0165136253,-0.4698271045\N,-2.6174923205,-3.3797784932,-0
.6252910544\H,-2.530221582,-4.3595268926,-0.8774960696\H,-3.5508498861
,-3.0130443148,-0.4677751205\C,1.5678323961,2.6084639383,-0.5356156003
\C,0.2423444385,3.2418065139,-0.6786996582\C,-0.0080820152,4.488983745
5,-0.0753617155\H,0.7569370186,4.9664573274,0.5290695006\C,-1.25596129
1,5.0946338844,-0.2200028036\H,-1.4494304161,6.0468604996,0.2638158117
\C,-2.2536762645,4.475327746,-0.9798380973\H,-3.2223683566,4.95204452,
-1.0953820958\C,-2.0052568312,3.2407008938,-1.5893494199\H,-2.77441939
53,2.7624912585,-2.1876058531\C,-0.7683091495,2.6177410089,-1.43146715
14\H,-0.5739089123,1.6660200448,-1.9133586916\C,-1.5551032949,-2.60289
59789,-0.5367578104\C,-0.229419445,-3.2349436228,-0.6832553276\C,0.779
0112747,-2.6095533028,-1.4378689683\H,0.5829357811,-1.6574069936,-1.91
82051661\C,2.0157862722,-3.2318045235,-1.5997127642\H,2.7831192216,-2.
7526289701,-2.1995475866\C,2.2663159345,-4.4670098837,-0.9922365876\H,
3.2348708218,-4.9432183809,-1.1109904708\C,1.2709074957,-5.0875382661,
-0.2303673786\H,1.4660770127,-6.0402208201,0.2518556366\C,0.0231347731
,-4.4826104788,-0.0818042982\H,-0.740185697,-4.9610568375,0.5240064464
\\Version=EM64L-G09RevB.01\HF=-2971.3060102\S2=2.500153\S2-1=0.\S2A=4.
920536\RMSD=0.000e+00\RMSF=3.230e-05\Dipole=0.0011491,-0.0002525,-1.88
04692\Quadrupole=-2.8549345,39.9709096,-37.1159751,24.0832767,-0.03821
2,0.03213\PG=Unknown\\@
```


Conformation c2


```
1\1\GINC-NODE164\FOpt\UB3LYP\6-31+G(d,p)\C14H14Fe1N4O2S2\SDS\11-Mar-20
21\0\\#N UB3LYP/6-31+G(d,p) Int(Grid=UltraFine) SCF(QC,Tight) SCRF=(Re
ad,Solvent=Water,SMD) Opt=(Redun) Freq(NoRaman) Symmetry(None) test\\[
Fe(NO)2(TBA)2]+ conf2 opt water\\1,2\Fe,0.0208120028,-0.0059483921,1.4
477972499\S,1.7373518805,0.9842814799,0.1021327635\S,-1.7317390279,-0.
9888181439,0.1381098829\O,-1.2871056119,1.8636004348,3.2425399156\O,1.
4271348748,-1.856865173,3.1861863771\N,-0.8222446938,1.1890133499,2.39
73871944\N,0.9141665961,-1.1970396899,2.3570948399\N,0.0576811782,3.00
6320849,-0.44460323\H,-0.154114292,3.9350138087,-0.7955928492\H,-0.693
45104,2.5018741572,0.0118731302\N,-0.0592382199,-3.0110192552,-0.42886
72589\H,0.1491769447,-3.9376057979,-0.7872183745\H,0.6933037521,-2.514
1191036,0.0336113669\C,1.2691992041,2.5011498589,-0.5648027436\C,2.265
4179268,3.2742458391,-1.3388337359\C,1.8590213323,4.0110010299,-2.4686
494995\H,0.8242568492,3.9892995945,-2.7960590049\C,2.7951231033,4.7389
872261,-3.2019169537\H,2.4759624659,5.2903874937,-4.0807133858\C,4.138
1744113,4.7511596143,-2.8119971765\H,4.8641480531,5.3224187492,-3.3826
160348\C,4.5468052824,4.0234448629,-1.6893921234\H,5.5871045466,4.0355
698337,-1.3798227757\C,3.6204762025,3.2799676154,-0.9601078296\H,3.941
3090964,2.7252346472,-0.0854645414\C,-1.2692575517,-2.5009969799,-0.54
44133874\C,-2.2667952591,-3.2660234883,-1.3246173256\C,-3.624243162,-3
.2625553469,-0.9546229137\H,-3.94725125,-2.7047066529,-0.0827648936\C,
-4.5506346177,-4.0012686618,-1.6887315999\H,-5.5929833831,-4.006065452
6,-1.3859875965\C,-4.1395085493,-4.7340960815,-2.8070996139\H,-4.86526
08065,-5.3024492939,-3.3808838127\C,-2.7940421391,-4.7308264449,-3.188
6379249\H,-2.4724470603,-5.2862402726,-4.0640163103\C,-1.8583081307,-4
.0068882703,-2.4511031766\H,-0.8217733549,-3.992446997,-2.7731694432\\
Version=EM64L-G09RevB.01\HF=-2971.3114351\S2=2.423699\S2-1=0.\S2A=4.58
9428\RMSD=0.000e+00\RMSF=7.989e-06\Dipole=-0.0463566,-0.0010353,-2.211
176\Quadrupole=-7.9654114,29.198115,-21.2327036,18.9906235,-0.0017338,
-0.0808459\PG=Unknown\\@
```


Conformation c3


```
1\1\GINC-NODE160\FOpt\UB3LYP\6-31+G(d,p)\C14H14Fe1N4O2S2\SDS\12-Mar-20
21\0\\#N UB3LYP/6-31+G(d,p) Int(Grid=UltraFine) SCF(QC,Tight) SCRF=(Re
ad,Solvent=Water,SMD) Opt=(Redun) Freq(NoRaman) Symmetry(None) test\\[
Fe(NO)2(TBA)2]+ conf3 opt water\\1,2\Fe,0.1108211922,-0.0192037982,2.0
63408332\S,1.9061407169,0.818700181,0.7064461536\S,-1.6053894922,-0.62
14018092,0.5082929434\O,-1.193196431,1.847164264,3.8768487659\O,1.3664
544332,-2.1074317761,3.6517880759\N,-0.7098092522,1.1727080269,3.04090
```

06278\N,0.9093555239,-1.3342492876,2.8899281875\N,1.7271575948,2.26419
33595,-1.4727479304\H,1.5284037536,3.0950246602,-2.0219921189\H,2.2537
249778,1.5188719716,-1.917440212\N,-1.4131852093,-2.0337781188,-1.6914
085513\H,-1.2698434522,-2.8724568833,-2.2461597819\H,-1.8090214488,-1.
2224785652,-2.1558862986\C,1.348161292,2.1721697352,-0.2130509877\C,0.
5089838515,3.256769304,0.3310510688\C,-0.4919728183,3.8401855765,-0.46
95687971\H,-0.6857389529,3.4580765505,-1.4670621518\C,-1.2656461002,4.
8856015625,0.0331241361\H,-2.0479621894,5.3185277009,-0.5823734505\C,-
1.0356967431,5.3703272455,1.3248775197\H,-1.6349932773,6.1892132708,1.
7112826416\C,-0.03653262,4.7989964816,2.1203526496\H,0.14836181,5.1784
241436,3.120343873\C,0.7265139429,3.7390362903,1.6337775947\H,1.504385
4227,3.3008382309,2.2493824098\C,-1.1321432696,-2.0121082074,-0.403290
4371\C,-0.4734454496,-3.198939913,0.1748146838\C,-0.851085293,-3.67775
80094,1.4418520095\H,-1.6208610447,-3.1634752732,2.0066105241\C,-0.257
7160537,-4.8291655694,1.9562460219\H,-0.5658953451,-5.2035113045,2.927
3526194\C,0.7310936031,-5.4977456224,1.226291128\H,1.1987725046,-6.388
3378335,1.6351070012\C,1.1209594534,-5.0175955349,-0.028330674\H,1.896
8259334,-5.5264327771,-0.5916399172\C,0.5163019564,-3.8794874474,-0.56
04611279\H,0.8339440332,-3.5022798787,-1.5276606399\\Version=EM64L-G09
RevB.01\HF=-2971.3054592\S2=2.482448\S2-1=0.\S2A=4.842251\RMSD=0.000e+
00\RMSF=1.772e-05\Dipole=0.0214048,0.103563,-5.188386\Quadrupole=-20.3
390742,26.2220199,-5.8829457,2.265167,-1.441429,0.1626934\PG=Unknown\\
@

Conformation c5

1\1\GINC-NODE168\FOpt\UB3LYP\6-31+G(d,p)\C14H14Fe1N4O2S2\SDS\29-Apr-20
21\0\\#N UB3LYP/6-31+G(d,p) Int(Grid=UltraFine) SCF(QC,Tight) SCRF=(Re
ad,Solvent=Water,SMD) Opt=(Redun) Freq(NoRaman) Symmetry(None) test\\[
Fe(NO)2(TBA)2]+ conf5 opt water\\1,2\Fe,-0.1883091968,0.3101108071,1.7
294273549\N,0.5896642781,-0.8096594064,2.8195907308\N,-1.0480745213,1.
6180489524,2.5010395318\O,1.0118725581,-1.3976598752,3.7478807176\O,-1
.5461835058,2.3794825907,3.247769551\S,1.6599292144,1.0533981101,0.403
4598013\C,1.2527048827,2.4020710859,-0.5879920685\N,0.0884266384,3.016
8385942,-0.5290825989\H,-0.1342557378,3.7760014808,-1.1651873444\H,-0.
6242658439,2.7493114394,0.1410679277\C,2.2642831761,2.9191464971,-1.53
60723628\C,3.1419103912,2.0394158326,-2.1960087118\C,4.0829245544,2.53
54055773,-3.0967367823\C,4.172649031,3.9107857505,-3.3352274616\C,3.31
19299754,4.7919962729,-2.6730314993\C,2.3573416125,4.3030567301,-1.782
2419475\H,3.07172735,0.9715010245,-2.0218591405\H,4.7451392992,1.84743
29264,-3.6128253234\H,4.9121408846,4.294504853,-4.0315483201\H,3.38652
38976,5.861337028,-2.8438666043\H,1.7134742316,5.0009355208,-1.2562310
379\S,-1.8920938762,-0.6976263876,0.3814214738\C,-1.3845681849,-2.2053
429781,-0.2807332696\N,-0.2510458035,-2.792037213,0.0468615443\H,0.040
3383753,-3.6547905102,-0.402017254\H,0.3695403881,-2.4006962668,0.7465
333417\C,-2.2695653956,-2.8974949093,-1.2434757006\C,-3.030897157,-2.1
643155211,-2.1719386821\C,-3.8475801132,-2.8283686478,-3.0858113889\C,
-3.9296384924,-4.2246776341,-3.0719433845\C,-3.1859946961,-4.958880940
6,-2.1425059649\C,-2.3541660911,-4.3036430657,-1.2357315758\H,-2.96452
34561,-1.0822863956,-2.1939469703\H,-4.4181996122,-2.2551989145,-3.809
7127841\H,-4.5729931672,-4.7382838649,-3.7799364619\H,-3.2570394136,-6
.0417297751,-2.1179019959\H,-1.8022665857,-4.8846062619,-0.5034794297\
\Version=EM64L-G09RevB.01\HF=-2971.3111331\S2=2.425765\S2-1=0.\S2A=4.5
96433\RMSD=0.000e+00\RMSF=2.701e-05\Dipole=0.114262,-0.2407609,-2.3464
126\Quadrupole=-14.3696422,33.0577516,-18.6881094,14.6871652,-0.645673
5,-5.0990661\PG=Unknown\\@

Conformation c7

1\1\GINC-NODE166\FOpt\UB3LYP\6-31+G(d,p)\C14H14Fe1N4O2S2\SDS\16-Mar-20
21\0\\#N UB3LYP/6-31+G(d,p) Int(Grid=UltraFine) SCF(QC,Tight) SCRF=(Re
ad,Solvent=Water,SMD) Opt=(Redun) Freq(NoRaman) Symmetry(None) test\\[
Fe(NO)2(TBA)2]+ conf7 g+ g- g+ opt water\\1,2\Fe,0.0069904626,0.004403
6644,2.2242567449\S,1.9044419693,1.4850025597,1.5061267393\S,-1.890430
2952,-1.4766400177,1.5066584341\O,-1.3405662165,1.4689742208,4.3271398
004\O,1.3544883675,-1.457835125,4.3283227825\N,-0.9504719117,1.0383226
487,3.2987830447\N,0.9648257365,-1.0291970822,3.2989181112\N,2.6080437
298,2.6319437564,-0.7472529745\H,2.4525466511,3.1131558544,-1.62715201
93\H,3.5635467166,2.5497167327,-0.4158106489\N,-2.5949482186,-2.625184
4956,-0.7455054711\H,-2.4399769037,-3.1071579077,-1.62508775\H,-3.5502
818811,-2.5424331678,-0.4137676082\C,1.5951368171,2.1596295751,-0.0405
643367\C,0.2488140013,2.2466600508,-0.6493734581\C,0.0739063893,1.9330
581176,-2.0099860861\H,0.9152453432,1.5869986388,-2.6020478288\C,-1.19
1186627,2.0284109462,-2.5901841825\H,-1.323055521,1.7669518052,-3.6355
036803\C,-2.2833002079,2.4566639407,-1.8288193951\H,-3.2653790044,2.53
71981648,-2.2850255042\C,-2.1105904216,2.780644095,-0.4788357107\H,-2.
9535163282,3.1241736474,0.1126678402\C,-0.8537510086,2.6660435288,0.11
45887084\H,-0.7197834811,2.9318954084,1.1570077237\C,-1.5816201045,-2.
1522845363,-0.0397443544\C,-0.2357166046,-2.2397793174,-0.6492274169\C
,0.8676023489,-2.6574675273,0.1146038899\H,0.7345241203,-2.9217606957,

1.1575370247\C,2.1240886741,-2.7725355077,-0.479470713\H,2.967526014,-
3.1148128956,0.1120269844\C,2.2957626335,-2.450683432,-1.8301021312\H,
3.277555861,-2.5315888783,-2.2868535147\C,1.2029459567,-2.0240452814,-
2.5913714844\H,1.3339909225,-1.7641613673,-3.6371885132\C,-0.061769352
6,-1.9282839372,-2.0104672588\H,-0.9034866266,-1.5834261837,-2.6027027
874\\Version=EM64L-G09RevB.01\HF=-2971.3013108\S2=2.627169\S2-1=0.\S2A
=5.511142\RMSD=0.000e+00\RMSF=1.316e-05\Dipole=-0.0003643,-0.0010605,-
5.3055567\Quadrupole=10.3861742,3.9986174,-14.3847916,14.2192881,-0.06
4378,-0.021696\PG=Unknown\\@

Conformation c9

1\1\GINC-NODE148\FOpt\UB3LYP\6-31+G(d,p)\C14H14Fe1N4O2S2\SDS\13-Mar-20
21\0\\#N UB3LYP/6-31+G(d,p) Int(Grid=UltraFine) SCF(QC,Tight) SCRF=(Re
ad,Solvent=Water,SMD) Opt=(Redun) Freq(NoRaman) Symmetry(None) test\\[
Fe(NO)2(TBA)2]+ conf9 tr g- g- opt water\\1,2\Fe,0.000457421,0.0129255
782,1.9941948942\S,0.5969484404,1.7206479776,0.3987004929\S,-0.5997167
369,-1.6823778388,0.3873565526\O,-2.3045035358,0.830563768,3.584452961
8\O,2.2995739935,-0.8269357937,3.5815859066\N,-1.431968997,0.534360665
8,2.8499429323\N,1.4310238097,-0.5189092004,2.8471740641\N,2.924528953
3,2.6644264639,-0.3541070554\H,3.8310810951,3.1029083772,-0.2248132877
\H,2.5764657568,2.569274432,-1.3027574528\N,-2.9301341818,-2.620841145
1,-0.3617232072\H,-3.8345370427,-3.0639119861,-0.2330904528\H,-2.58968
36745,-2.5088082566,-1.311280833\C,2.2090137359,2.2633653749,0.6803614
245\C,2.8255645703,2.3471633586,2.018873182\C,4.1890158453,2.038264171
8,2.1861041322\H,4.7816358726,1.7000893969,1.3418245158\C,4.7729050911
,2.1224736568,3.4497327774\H,5.8196208703,1.8639107605,3.5753548936\C,
4.0123638203,2.532091235,4.5496475794\H,4.4714767204,2.6017304309,5.53
11699395\C,2.6592291018,2.8483841398,4.3865693421\H,2.0679652433,3.173
7058731,5.2367543357\C,2.0626837808,2.7462201983,3.1312840322\H,1.0148
722547,2.9960029443,3.0061433238\C,-2.2082967449,-2.2330142268,0.67331
88951\C,-2.8136202968,-2.3389236555,2.0152393506\C,-2.0391871687,-2.74
94259973,3.1154730612\H,-0.9911363958,-2.991795666,2.9782928661\C,-2.6
247481448,-2.8729894353,4.3740002159\H,-2.0246781512,-3.2072695053,5.2
144843271\C,-3.9782288451,-2.566809174,4.5528241959\H,-4.4285613275,-2
.6535801665,5.5370427864\C,-4.7502085367,-2.145765406,3.4652654764\H,-
5.7972746542,-1.8949800732,3.6031672225\C,-4.1773725232,-2.0400857283,
2.1981565166\H,-4.7791881191,-1.6928206234,1.3640957974\\Version=EM64L
-G09RevB.01\HF=-2971.3063597\S2=2.518928\S2-1=0.\S2A=5.001059\RMSD=0.0
00e+00\RMSF=1.004e-05\Dipole=-0.0037812,0.0129038,-2.5235045\Quadrupol
e=22.6628943,-12.6414487,-10.0214457,40.4506825,0.0938127,-0.1688215\P
G=Unknown\\@

Conformation c12

1\1\GINC-NODE160\FOpt\UB3LYP\6-31+G(d,p)\C14H14Fe1N4O2S2\SDS\14-Mar-20
21\0\\#N UB3LYP/6-31+G(d,p) Int(Grid=UltraFine) SCF(QC,Tight) SCRF=(Re
ad,Solvent=Water,SMD) Opt=(Redun) Freq(NoRaman) Symmetry(None) test\\[
Fe(NO)2(TBA)2]+ conf12 tr tr g+ opt water\\1,2\Fe,0.0834795336,-0.1261
563188,1.998937935\S,1.7890805248,0.3897269379,0.3526100914\S,-1.58150
58822,-0.7515029628,0.3438636284\O,-0.7276529635,2.2364578944,3.508893
7337\O,0.8156794732,-2.3439831639,3.7491561724\N,-0.4159326847,1.34999
1456,2.7979580825\N,0.5418540663,-1.523475555,2.9487041743\N,3.2003567
653,1.3097715497,2.4263738789\H,3.9303031998,1.87478204,2.8486308642\H
,2.5771677438,0.8095068964,3.0514439753\N,-3.1549244685,-1.3493101568,
2.4210935976\H,-3.9687898456,-1.779478312,2.8489207834\H,-2.4775526668
,-0.9155910161,3.0391988496\C,3.0658176481,1.248133115,1.1141643855\C,
4.0871067265,1.9183891465,0.2801342165\C,5.4301612135,1.9558355215,0.7
035027414\H,5.7323435324,1.4609574249,1.621145918\C,6.3918613438,2.593
4128194,-0.0789397742\H,7.4270225525,2.603896573,0.2475320503\C,6.0261
700974,3.2085635824,-1.2806690131\H,6.7768840846,3.7082745331,-1.88538
87581\C,4.6937894324,3.1757258732,-1.7053353316\H,4.4046497494,3.65707
28074,-2.6342744445\C,3.7285872619,2.5276383753,-0.9363055048\H,2.6951
147087,2.5146411099,-1.264470423\C,-2.9804773653,-1.3798824439,1.11255
48885\C,-4.0624443156,-1.9492160534,0.2781963486\C,-3.7583714801,-2.67
954279,-0.8850181642\H,-2.724175109,-2.8422228502,-1.1676187275\C,-4.7
83537234,-3.2190594519,-1.6602169881\H,-4.538507353,-3.7936954141,-2.5
479122355\C,-6.1197168359,-3.0221448305,-1.2959493575\H,-6.9161430906,
-3.4371166729,-1.9062845132\C,-6.4292505811,-2.2871692789,-0.147082330
8\H,-7.4647003643,-2.1202314264,0.132595253\C,-5.4093223376,-1.7568256
385,0.6420860942\H,-5.663231073,-1.1672713668,1.5173487394\\Version=EM
64L-G09RevB.01\HF=-2971.3089482\S2=2.508057\S2-1=0.\S2A=4.964414\RMSD=
0.000e+00\RMSF=1.362e-05\Dipole=-0.068918,0.2525712,2.4247502\Quadrupo
le=42.1983938,-30.5574178,-11.640976,38.6800897,0.6627577,0.4943553\PG
=Unknown\\@

Conformation c13

1\1\GINC-NODE173\FOpt\UB3LYP\6-31+G(d,p)\C14H14Fe1N4O2S2\SDS\16-Mar-20
21\0\\#N UB3LYP/6-31+G(d,p) Int(Grid=UltraFine) SCF(QC,Tight) SCRF=(Re
ad,Solvent=Water,SMD) Opt=(Redun) Freq(NoRaman) Symmetry(None) test\\[

Fe(NO)2(TBA)2]+ conf13 symmetry Cs g+ g+ g+ opt water\\1,2\Fe,-0.22469
81402,0.4169305193,1.2585275285\S,1.6163387812,0.9167541447,-0.2012927
491\S,-1.7063790192,-0.7184925768,-0.256485081\O,-1.4489988607,2.79759
68253,2.4037607912\O,0.7664887555,-1.6011567496,3.1285469061\N,-0.9722
564434,1.8685492218,1.8631671079\N,0.4009626728,-0.8473812497,2.298275
5746\N,1.8624567902,2.9586375208,-1.8325635122\H,1.9956259083,3.934667
1603,-2.0806214683\H,1.9646823782,2.2663247654,-2.5676981361\N,-3.4957
422762,0.3674403826,-1.8401208761\H,-4.3637872884,0.8427078136,-2.0690
175046\H,-2.9882758258,-0.0788472786,-2.5974264919\C,1.6066278141,2.60
02327423,-0.5892546026\C,1.3847385098,3.6685581239,0.4043436034\C,0.66
90625558,4.8267042646,0.0454223038\H,0.2372301791,4.9193090039,-0.9462
883883\C,0.4764769205,5.8450961554,0.9784001533\H,-0.0916438654,6.7271
792356,0.7001582374\C,1.0092710337,5.7274292229,2.2661532562\H,0.86315
14274,6.5248128416,2.9885678621\C,1.7256546607,4.5806082915,2.62558520
33\H,2.1446869864,4.4886776559,3.6226846024\C,1.9043626046,3.548774491
2,1.7060524556\H,2.4632624063,2.662381626,1.9852642354\C,-3.061974483,
0.2978817136,-0.596467038\C,-3.7988402917,1.0543270647,0.4344623723\C,
-4.0087197663,0.5013323903,1.7106968278\H,-3.6244032407,-0.4860775099,
1.9415648149\C,-4.7336323979,1.2099059213,2.6669606988\H,-4.9062827281
,0.769548987,3.6439594562\C,-5.2327268869,2.4830223202,2.3706843693\H,
-5.7863614157,3.0373106072,3.1225712913\C,-5.0149816266,3.0440434502,1
.1081316459\H,-5.3890952628,4.0370786885,0.8796184809\C,-4.3102559072,
2.3323453357,0.1378166518\H,-4.1286625413,2.7824445203,-0.8331689754\\
Version=EM64L-G09RevB.01\HF=-2971.3063583\S2=2.497705\S2-1=0.\S2A=4.90
5032\RMSD=0.000e+00\RMSF=2.602e-05\Dipole=-1.334442,2.7589934,-4.04290
43\Quadrupole=4.0028554,6.2308206,-10.233676,-2.0931587,1.642694,-4.71
46895\PG=Unknown\\@

Conformation c15

1\1\GINC-NODE169\FOpt\UB3LYP\6-31+G(d,p)\C14H14Fe1N4O2S2\SDS\17-Mar-20
21\0\\#N UB3LYP/6-31+G(d,p) Int(Grid=UltraFine) SCF(QC,Tight) SCRF=(Re
ad,Solvent=Water,SMD) Opt=(Redun) Freq(NoRaman) Symmetry(None) test\\[
Fe(NO)2(TBA)2]+ conf15 symmetry Cs g+ tr g+ opt water\\1,2\Fe,-0.01913
57008,0.0431766488,1.3176670697\S,1.6830980075,1.0549990169,-0.0504041
864\S,-1.8185811544,-0.7240391125,-0.0741979191\O,-0.733635147,1.27219
73111,3.8461107245\O,1.25365369,-2.4544719702,2.0972561354\N,-0.527722
907,0.9492374363,2.7291294606\N,0.7606036481,-1.4773019665,1.672869702
9\N,0.4029019324,3.3949082063,0.2219703294\H,0.2260527313,4.3655838018
,-0.0168899205\H,-0.2088043596,2.967566222,0.9083796952\N,-2.985344873
1,1.6509914393,0.3279254139\H,-3.6706492073,2.3732470465,0.1297725697\
H,-2.2919730727,1.8466397259,1.0416123681\C,1.3824945722,2.7234999681,
-0.3503641375\C,2.2740611518,3.4584138908,-1.2751684206\C,2.5996890665
,4.8046289889,-1.01842386\H,2.2338971427,5.2989320837,-0.124140311 4\C,
3.435368141,5.500814933,-1.8905756499\H,3.6935014025,6.5328750771,-1.6
749786443\C,3.9437654032,4.8704037736,-3.0307043056\H,4.5906734493,5.4
16443921,-3.710616845\C,3.6219188051,3.5343787122,-3.2917201907\H,4.00
95000101,3.0429787322,-4.1785219971\C,2.7998841412,2.8261488025,-2.416
5550047\H,2.5464141117,1.7937136774,-2.6299759848\C,-3.0007607438,0.50
28995905,-0.319242757\C,-4.1022514259,0.2587330527,-1.2755703699\C,-3.
8764726468,-0.4772376788,-2.4531973858\H,-2.8860888285,-0.8611653265,-
2.6710251757\C,-4.9167723799,-0.6870183899,-3.3568804324\H,-4.72893370
88,-1.2422273242,-4.2704725018\C,-6.1942101315,-0.1841189812,-3.088974
0635\H,-7.0043934222,-0.3564908883,-3.7910168958\C,-6.4281658539,0.538
1979453,-1.9143308328\H,-7.4209244124,0.9184165254,-1.695041592\C,-5.3
897715837,0.7659274057,-1.0124011559\H,-5.5920567352,1.306022003,-0.09
28623123\\Version=EM64L-G09RevB.01\HF=-2971.3108503\S2=2.44903\S2-1=0.
\S2A=4.700505\RMSD=0.000e+00\RMSF=1.106e-05\Dipole=-2.2350813,4.31568,
-0.6540729\Quadrupole=11.2742544,11.5041956,-22.7784499,-0.8601164,-0.
25062,-0.444446\PG=Unknown\\@

Conformation c16

1\1\GINC-NODE153\FOpt\UB3LYP\6-31+G(d,p)\C14H14Fe1N4O2S2\SDS\22-Mar-20
21\0\\#N UB3LYP/6-31+G(d,p) Int(Grid=UltraFine) SCF(QC,Tight) SCRF=(Re
ad,Solvent=Water,SMD) Opt=(Redun) Freq(NoRaman) Symmetry(None) test\\[
Fe(NO)2(TBA)2]+ conf16 symmetry Cs g+ tr g- opt water\\1,2\Fe,-0.09660
8317,0.2559600553,0.9374586669\S,1.5771540646,1.1899504069,-0.51894920
53\S,-1.9128595777,-0.6611560504,-0.3416194494\O,-0.7167111262,1.68952
21455,3.3760953101\O,1.2461545604,-2.1474028943,1.8773843734\N,-0.5566
827034,1.2693864996,2.2850195916\N,0.7174298689,-1.221301847,1.3853534
024\N,0.4966431076,3.6059008477,-0.1069090496\H,0.5108261646,4.6208935
6,-0.1182105563\H,-0.3015482018,3.156350766,0.3262452864\N,-3.18759655
54,1.674359456,-0.0365374748\H,-3.9986386218,2.2816720218,-0.097765423
3\H,-2.3678454813,2.0389875478,0.434783767\C,1.4917365469,2.9063131035
,-0.6158715971\C,2.5666735216,3.6411318113,-1.3171277287\C,2.263458655
2,4.8109909456,-2.0411440812\H,1.2379775492,5.1568864001,-2.1220011618
\C,3.2753588823,5.5103137204,-2.6968957765\H,3.0292364244,6.4025340732
,-3.263958785 8\C,4.5978294853,5.0598171179,-2.6307076444\H,5.384631785
2,5.6089864055,-3.1389619771\C,4.9053608842,3.8990510779,-1.9132954951

```
\H,5.9311668236,3.5491062866,-1.8547806491\C,3.8972763487,3.186317879,
-1.2663685769\H,4.1438587552,2.2932869421,-0.70316446\C,-3.2172420181,
0.4480751738,-0.5194535711\C,-4.4252974077,0.0255613885,-1.2614534685\
C,-4.8845822614,-1.3021912454,-1.1864980761\H,-4.3589109976,-2.0237934
722,-0.5711852076\C,-6.0326510911,-1.6853070618,-1.8777740456\H,-6.388
0215944,-2.7080269537,-1.8008291492\C,-6.7235699772,-0.7573989252,-2.6
641282244\H,-7.6133424988,-1.0607796221,-3.2074512113\C,-6.2649966132,
0.5607200937,-2.7540333934\H,-6.7888294694,1.2809091466,-3.3746592698\
C,-5.1262847539,0.9561286405,-2.0535325383\H,-4.7671881606,1.975279559
,-2.1544441496\\Version=EM64L-G09RevB.01\HF=-2971.3110973\S2=2.438964\
S2-1=0.\S2A=4.658453\RMSD=0.000e+00\RMSF=1.926e-05\Dipole=-2.1928661,4
.2920548,-0.6108847\Quadrupole=19.992985,15.7021778,-35.6951628,4.0770
976,5.709494,-11.8128159\PG=Unknown\\@
```


Conformation c19


```
1\1\GINC-NODE174\FOpt\UB3LYP\6-31+G(d,p)\C14H14Fe1N4O2S2\SDS\20-Mar-20
21\0\\#N UB3LYP/6-31+G(d,p) Int(Grid=UltraFine) SCF(QC,Tight) SCRF=(Re
ad,Solvent=Water,SMD) Opt=(Redun) Freq(NoRaman) Symmetry(None) test\\[
Fe(NO)2(TBA)2]+ conf19 symmetry Cs tr tr g+ opt water\\1,2\Fe,-1.43551
52887,-0.1508913594,0.0028509805\S,0.0766060892,0.5257035898,-1.782564
1681\S,0.0791464962,0.5213579588,1.7884387527\O,-1.8643315367,-3.04041
77145,-0.0003950298\O,-3.9046577397,1.4144497273,0.0022565018\N,-1.577
6913939,-1.897637132,0.0009384243\N,-2.8548823409,0.8784826654,0.00306
23717\N,-1.7568716592,-0.5086078162,-3.4349292272\H,-2.0660598426,-0.8
651175651,-4.3335555289\H,-2.4039352854,-0.5842635696,-2.6568748259\N,
-1.7533192642,-0.5152876718,3.4400232884\H,-2.0625077663,-0.8733253166
,4.3380364668\H,-2.4003798546,-0.5889080666,2.661815712\C,-0.546844933
,0.0016718423,-3.2927325088\C,0.2878167377,0.1495902707,-4.5059014833\
C,-0.3131920305,0.4593687054,-5.7413370051\H,-1.3840403658,0.623910646
9,-5.8058369801\C,0.4715571125,0.5974911043,-6.8854920939\H,0.00112290
23,0.8515989398,-7.830133726\C,1.8568297373,0.4186732787,-6.8140484514
\H,2.4649236822,0.5238370346,-7.7074373635\C,2.4589255409,0.1085803486
,-5.5901001064\H,3.5329178946,-0.0372855211,-5.5309607764\C,1.68294970
52,-0.0177349912,-4.4390941038\H,2.1537240839,-0.2686449483,-3.4950740
12\C,-0.5430718439,-0.0054198537,3.2981439465\C,0.2928532934,0.1391117
707,4.5108343453\C,1.6878420892,-0.0285528384,4.4418672406\H,2.1573427
204,-0.2769327606,3.4965489583\C,2.4653061038,0.094074379,5.5922681321
\H,3.5391600382,-0.0520894084,5.5313933878\C,1.8648435332,0.4009430369
,6.8178292762\H,2.4740623699,0.5032573277,7.7107821294\C,0.4797296784,
0.5801315105,6.8914709418\H,0.010579265,0.8316839876,7.8374309073\C,-0
.3065151326,0.44565018,5.7479000802\H,-1.3772367957,0.6102942284,5.814
267547\\Version=EM64L-G09RevB.01\HF=-2971.3091537\S2=2.524492\S2-1=0.\
S2A=5.036834\RMSD=0.000e+00\RMSF=9.170e-06\Dipole=-2.1196942,-1.344409
,0.0004477\Quadrupole=-14.5472332,-47.1631047,61.710338,4.7298055,0.02
93281,-0.1313139\PG=Unknown\\@
```


Conformation c20


```
1\1\GINC-NODE153\FOpt\UB3LYP\6-31+G(d,p)\C14H14Fe1N4O2S2\SDS\20-Mar-20
21\0\\#N UB3LYP/6-31+G(d,p) Int(Grid=UltraFine) SCF(QC,Tight) SCRF=(Re
ad,Solvent=Water,SMD) Opt=(Redun) Freq(NoRaman) Symmetry(None) test\\[
Fe(NO)2(TBA)2]+ conf20 symmetry Cs g+ g- g+ opt water\\1,2\Fe,-0.04876
05371,0.215292694,0.6896281774\S,1.6837587102,1.2105979294,-0.64423401
43\S,-1.7713358146,-0.2955981956,-0.9074219014\O,-0.6100952251,1.66047
59728,3.1450241064\O,1.226985423,-2.2760333729,1.4930258905\N,-0.48397
09774,1.2115654814,2.0604669391\N,0.7182100206,-1.3093749018,1.0609000
473\N,0.1898396623,3.433802266,-0.6727354353\H,-0.057644798,4.35687626
17,-1.0148721596\H,-0.4248064071,3.0128014764,0.0154533816\N,-3.492088
4053,1.1869377344,0.5106705976\H,-4.3327418442,1.7430829785,0.62981392
29\H,-2.8726287515,1.1048504996,1.3084237116\C,1.2631196437,2.81031972
82,-1.1181970145\C,2.1502505894,3.5373559087,-2.0533335983\C,2.3376750
889,4.9263502208,-1.9125790006\H,1.8630494526,5.4667751282,-1.09965910
56\C,3.1730983499,5.6118823117,-2.793582326\H,3.3243187143,6.679268296
9,-2.6667532242\C,3.8180399779,4.9269125478,-3.8283685098\H,4.46467916
02,5.464312910 4,-4.5153987514\C,3.6332776604,3.5481546381,-3.974672232
1\H,4.1268484842,3.0134696999,-4.7801079915\C,2.8123419068,2.852403024
1,-3.0887061633\H,2.6657201893,1.7852876544,-3.2133022746\C,-3.2225723
075,0.592265613,-0.6346622222\C,-4.2372828251,0.6605786481,-1.70816167
23\C,-3.8470693443,0.7186660583,-3.058836885\H,-2.7946094948,0.7173948
6,-3.3194460687\C,-4.8108964043,0.8089407974,-4.0617782036\H,-4.499803
9501,0.8695286037,-5.0999035289\C,-6.1703355723,0.824947236,-3.7330400
711\H,-6.918226629,0.8890065603,-4.5175339893\C,-6.5660504856,0.753663
2507,-2.3933254746\H,-7.6199080773,0.7509299083,-2.1334419754\C,-5.608
4068232,0.6774066885,-1.3835827981\H,-5.9306122042,0.5980876776,-0.350
2377326\\Version=EM64L-G09RevB.01\HF=-2971.3099454\S2=2.454888\S2-1=0.
\S2A=4.725504\RMSD=0.000e+00\RMSF=1.039e-05\Dipole=-2.9998864,3.404793
5,-0.0396766\Quadrupole=15.8391774,2.5458933,-18.3850707,-3.1009172,-4
.2720757,-14.320464\PG=Unknown\\@
```


Conformation c21

1\1\GINC-NODE173\FOpt\UB3LYP\6-31+G(d,p)\C14H14Fe1N4O2S2\SDS\21-Mar-20
21\0\\#N UB3LYP/6-31+G(d,p) Int(Grid=UltraFine) SCF(QC,Tight) SCRF=(Re
ad,Solvent=Water,SMD) Opt=(Redun) Freq(NoRaman) Symmetry(None) test\\[
Fe(NO)2(TBA)2]+ conf21 symmetry Cs g+ g- g- opt water\\1,2\Fe,-0.15116
18074,0.5884696661,0.1031741183\S,1.6028624625,1.3774010362,-1.3077196
706\S,-2.0045559483,0.4367221534,-1.4520408082\O,-0.3839688042,2.10671
96571,2.5672761997\O,0.8044879823,-2.0824742937,0.7716009233\N,-0.3830
57073,1.6291843615,1.488358832\N,0.4098601938,-1.0384051673,0.40232036
54\N,0.6369160148,3.8594137631,-0.9842210463\H,0.6947783287,4.87079029
57,-1.0520478076\H,-0.1708358639,3.4737809025,-0.5086988936\N,-3.67658
11687,0.7917242067,0.608713831\H,-4.597030988,0.8180673403,1.035360062
6\H,-2.8770444494,0.9252006234,1.2166356425\C,1.5876768548,3.093201292
2,-1.4791853188\C,2.6769911013,3.7424352505,-2.2400428864\C,2.40912761
88,4.8866169467,-3.0174487903\H,1.3996924286,5.277883402,-3.0934318525
\C,3.4343380929,5.4994020959,-3.7362487141\H,3.2144881133,6.370729163,
-4.345047679\C,4.7349720622,4.9881840743,-3.6811472768\H,5.5317505115,
5.4694984933,-4.2400756345\C,5.0075887309,3.8539015629,-2.9093887494\H
,6.0166609488,3.4572526665,-2.8590998429\C,3.985753027,3.2270627893,-2
.1984464415\H,4.2059355605,2.3544174358,-1.5938075623\C,-3.5344785469,
0.5826674106,-0.6863128498\C,-4.7605740244,0.4886631962,-1.5104735303\
C,-4.8197171064,-0.3884482116,-2.6085726172\H,-3.9680788606,-1.0156047
286,-2.8478151532\C,-5.9824336384,-0.4750891521,-3.3725116122\H,-6.023
8887341,-1.1664852706,-4.208256408\C,-7.0891667166,0.3223313185,-3.063
0851879\H,-7.9904622224,0.2583483501,-3.6651853521\C,-7.0334939634,1.2
039260266,-1.9788998636\H,-7.8850975108,1.8338899649,-1.7419296142\C,-
5.8798332812,1.2850347727,-1.1996116295\H,-5.8416939267,1.991036746,-0
.3760262082\\Version=EM64L-G09RevB.01\HF=-2971.3097432\S2=2.461084\S2-
1=0.\S2A=4.754284\RMSD=0.000e+00\RMSF=1.076e-05\Dipole=-2.3150295,2.82
24861,0.8654366\Quadrupole=26.7893454,3.6461627,-30.4355081,6.3768357,
-6.1228717,-15.7207916\PG=Unknown\\@

Conformation c22

1\1\GINC-NODE145\FOpt\UB3LYP\6-31+G(d,p)\C14H14Fe1N4O2S2\SDS\16-Apr-20
21\0\\#N UB3LYP/6-31+G(d,p) Int(Grid=UltraFine) SCF(QC,Tight) SCRF=(Re
ad,Solvent=Water,SMD) Opt=(Redun) Freq(NoRaman) Symmetry(None) test\\[
Fe(NO)2(TBA)2]+ nonsym g+ tr g+ / tr tr g+ opt water\\1,2\Fe,-0.030511
7252,0.1950008043,2.0088552737\N,0.7824742968,-1.1479934889,2.77443988
53\N,-0.695413953,1.4368875879,3.0516133121\O,1.2910033211,-1.95514405
14,3.462225427\O,-1.0043072144,2.1275903469,3.9570043514\S,1.636207530
8,1.0196780726,0.510470092\C,1.1718444995,2.5170941914,-0.2085857759\N
,0.0824285218,3.1733518126,0.134919874\H,-0.1915906557,4.021981329,-0.
3502862023\H,-0.5265411635,2.8467834864,0.8772983874\C,2.0432649444,3.
1086035334,-1.2472205707\C,2.7347821398,2.2843125283,-2.1540066774\C,3
.5406917476,2.8511797631,-3.1398863602\C,3.6816175049,4.240418242,-3.2
211734166\C,3.0076689434,5.0650800866,-2.314608926\C,2.1868900704,4.50
73121557,-1.3355417213\H,2.6227427033,1.2070990989,-2.1020611676\H,4.0
571839593,2.2074716514,-3.8448742631\H,4.316496349,4.6786592663,-3.985
2461567\H,3.1252405803,6.142997834,-2.3641476777\H,1.6910768404,5.1588
606907,-0.6229633175\S,-1.6127731851,-0.6506544166,0.364562917\C,-2.77
49718818,-1.6511440272,1.1345147603\N,-2.879272103,-1.7436647366,2.447
9800486\H,-3.5328936976,-2.3927266117,2.8741439075\H,-2.2947502712,-1.
1926785644,3.0678111406\C,-3.7201907347,-2.43201489,0.3055229072\C,-3.
3035625424,-2.9857056241,-0.918812739\C,-4.1933835615,-3.7349896063,-1
.6868603553\C,-5.5090828909,-3.9267897773,-1.2524222252\C,-5.933142007
9,-3.3695840014,-0.0417539017\C,-5.0455018158,-2.6301667901,0.73922383
99\H,-2.2815654118,-2.8490124069,-1.2544971842\H,-3.8581877826,-4.1707
423739,-2.6227930811\H,-6.201757997,-4.5055753693,-1.8559454491\H,-6.9
569881336,-3.5039987248,0.2928295131\H,-5.3952563954,-2.1817403031,1.6
637551126\\Version=EM64L-G09RevB.01\HF=-2971.310345\S2=2.47595\S2-1=0.
\S2A=4.818435\RMSD=0.000e+00\RMSF=4.789e-05\Dipole=-2.3657086,0.985761
8,0.2137483\Quadrupole=3.5595631,11.2720487,-14.8316118,30.2453206,-22
.8628841,-11.7533649\PG=Unknown\\@

Conformation c23

1\1\GINC-NODE173\FOpt\UB3LYP\6-31+G(d,p)\C14H14Fe1N4O2S2\SDS\29-Apr-20
21\0\\#N UB3LYP/6-31+G(d,p) Int(Grid=UltraFine) SCF(QC,Tight) SCRF=(Re
ad,Solvent=Water,SMD) Opt=(Redun) Freq(NoRaman) Symmetry(None) test\\[
Fe(NO)2(TBA)2]+ conf23 g+ tr g- / tr tr g- opt water\\1,2\Fe,0.0227142
924,0.0857918981,1.7314873017\N,0.9559965132,-1.0760320299,2.643250302
1\N,-0.762482464,1.3700493157,2.6194720611\O,1.5418585594,-1.748177742
2,3.4112089061\O,-1.1365646816,2.1357511438,3.4345908641\S,1.619370903
3,0.9211038099,0.1735532576\C,1.1419713676,2.4309639806,-0.5113924454\
N,-0.0605317816,2.9474409224,-0.359946626\H,-0.2785262223,3.8702438622
,-0.7229356132\H,-0.799391584,2.4589027297,0.1334397732\C,2.117896127,
3.175593194,-1.3359264809\C,3.4870902914,3.1593533259,-1.0111812135\C,
4.3973112064,3.8797760287,-1.7824960176\C,3.9578449903,4.6077536585,-2
.8930998298\C,2.6002620804,4.6176063221,-3.229645433\C,1.6809947807,3.
9118114131,-2.4551815027\H,3.8326094787,2.6047884903,-0.1460045335\H,5

.4491818816,3.8739712473,-1.5147916272\H,4.6706910064,5.1619615991,-3.
4961175685\H,2.2564994638,5.1689385631,-4.0991429245\H,0.634526758,3.9
068824241,-2.7429907087\S,-1.4768285187,-1.1332805597,0.2539754659\C,-
2.8001047088,-1.7699426049,1.1429162807\N,-2.8600796939,-1.729410492,2
.4618156025\H,-3.6803856863,-2.0643382358,2.9564416787\H,-2.0968785234
,-1.3606579493,3.0190059998\C,-3.9134782635,-2.4208184638,0.4162457107
\C,-4.316988472,-1.9445137279,-0.8446023635\C,-5.3747458862,-2.5531473
112,-1.5180004045\C,-6.0302653224,-3.651537457,-0.952037008\C,-5.62670
17236,-4.138355281,0.2954416043\C,-4.5786202651,-3.5263397983,0.981956
5168\H,-3.8186774942,-1.0875448741,-1.2840246578\H,-5.6877907817,-2.16
80381576,-2.4833699087\H,-6.8492541314,-4.1280828713,-1.4822744803\H,-
6.1227709653,-4.9992214848,0.7325535999\H,-4.2607031397,-3.93193036,1.
9373424473\\Version=EM64L-G09RevB.01\HF=-2971.3107969\S2=2.464888\S2-1
=0.\S2A=4.771834\RMSD=0.000e+00\RMSF=3.475e-05\Dipole=-2.5750038,1.247
6332,0.1641389\Quadrupole=10.021516,5.021208,-15.042724,32.423155,-15.
2845089,-23.8124636\PG=Unknown\\@

Conformation c24, crystalline

1\1\GINC-NODE159\FOpt\UB3LYP\6-31+G(d,p)\C14H14Fe1N4O2S2\SDS\08-Mar-20
21\0\\#N UB3LYP/6-31+G(d,p) Int(Grid=UltraFine) SCF(QC,Tight) SCRF=(Re
ad,Solvent=Water,SMD) Opt=(Redun) Freq(NoRaman) Symmetry(None) test\\[
Fe(NO)2(TBA)2]+ crist struct opt water\\1,2\Fe,-0.6564082686,1.3141676
395,0.5413767172\S,1.1694874365,-0.2441444269,0.5385895807\S,-1.860672
1955,1.4239911402,-1.5298337699\O,0.1833012354,4.028559454,1.159606647
9\O,-2.3667842862,0.3658868157,2.6935113792\N,-0.064296313,2.943122686
9,0.7710429975\N,-1.6888150414,0.6472651651,1.7746248075\N,2.551057458
,1.517855535,-0.9359632092\H,3.4064789253,1.893131854,-1.3330064704\H,
1.7029388893,2.0549885388,-1.0747257184\N,-4.3098639224,0.6960798051,-
2.1100131343\H,-5.0912522616,0.0529074709,-2.1987595044\H,-4.393233154
2,1.6055882968,-2.5533264626\C,2.5603555803,0.3905283066,-0.2513246428
\C,3.8284778342,-0.3702668377,-0.1911524013\C,4.7066008024,-0.37166219
57,-1.2931445513\H,4.4443467612,0.1482641658,-2.2092910948\C,5.9049868
944,-1.0814505204,-1.2315568927\H,6.5677014895,-1.0887040711,-2.091190
9768\C,6.2471395496,-1.7860081234,-0.0727917202\H,7.1835522918,-2.3336
822557,-0.0263232997\C,5.378773347,-1.7894988684,1.0238160412\H,5.6417
604375,-2.3311942685,1.9269357392\C,4.1723881909,-1.0939817973,0.96553
72153\H,3.508507457,-1.0914225327,1.8226654371\C,-3.2119613722,0.34743
32986,-1.468560291\C,-3.2092095172,-0.9505826411,-0.7681409804\C,-2.07
99451899,-1.7867217741,-0.8209067172\H,-1.2034038459,-1.4767753742,-1.
3786598714\C,-2.1021469399,-3.0280563585,-0.1872548732\H,-1.2342913422
,-3.677103977,-0.2483389112\C,-3.236536889,-3.4343648378,0.5235833542\
H,-3.246104092,-4.397435861,1.0249746087\C,-4.3576864861,-2.6005807438
,0.5905751906\H,-5.2346316472,-2.9079705222,1.1515267354\C,-4.35238994
23,-1.3667766367,-0.0592854228\H,-5.2187188735,-0.7166455486,0.0106424
644\\Version=EM64L-G09RevB.01\HF=-2971.308696\S2=2.459872\S2-1=0.\S2A=
4.745145\RMSD=0.000e+00\RMSF=3.877e-05\Dipole=-0.838587,0.1207126,-2.8
183394\Quadrupole=47.6589877,-23.3808941,-24.2780936,1.7528096,6.79262
17,-10.8897215\PG=Unknown\\@

Conformational structures of complex **I** (complex ***2***) optimized by the B3LYP/6-31+G(d,p) method.

Conformation c1, crystalline

1\1\GINC-NODE153\FOpt\UB3LYP\6-31+G(d,p)\C7H7Fe1N3O5S3(1-,2)\SDS\04-Ap
r-2021\0\\#N UB3LYP/6-31+G(d,p) Int(Grid=UltraFine) SCF(QC,Tight) SCRF
=(Read,Solvent=Water,SMD) Opt=(Redun) Freq(NoRaman) test\\[Fe(NO)2(TBA
)(SSO3)]- conf1 crist opt water\\-1,2\Fe,-4.4830638608,-14.1284613354,
-3.7467950917\S,-4.3504272307,-13.6142614559,-1.3849901206\S,-6.693148
6903,-14.2361635212,-4.4431517447\S,-7.1067563316,-16.3138939509,-4.64
11008526\O,-3.2554688682,-11.7533668564,-4.9222284452\O,-3.0254053017,
-16.5752944752,-4.3636472043\O,-8.5991108646,-16.3956310365,-4.7660260
556\O,-6.6087916475,-17.0010923003,-3.3956032313\O,-6.4020395834,-16.8
122946131,-5.8671872028\N,-3.8157110967,-12.642387064,-4.3809814817\N,
-3.6733236904,-15.6424089893,-4.0392981909\N,-5.8199789795,-15.8081727
98,-0.9044927402\H,-6.3343654321,-16.417736006,-0.2764687445\H,-5.8876
940657,-16.0333674195,-1.9018310571\C,-5.1736631857,-14.7685380861,-0.
4167452009\C,-5.1514610602,-14.6006380355,1.0582202967\C,-5.2233676304
,-13.3198371664,1.6344510045\H,-5.3093305673,-12.4449016081,0.99958288
27\C,-5.2118208059,-13.1734341585,3.0210256504\H,-5.2840427401,-12.181
2196398,3.4555583775\C,-5.1093834894,-14.2975332465,3.8472762037\H,-5.
0915898535,-14.1795022407,4.9265496954\C,-5.0261250945,-15.5731137652,
3.2806906087\H,-4.9334311388,-16.4486525511,3.9157815566\C,-5.05399159
7,-15.7288285951,1.8945524376\H,-4.9665071942,-16.7232690855,1.4678586
505\\Version=EM64L-G09RevB.01\State=2-A\HF=-3269.7227739\S2=2.4889\S2-

```
1=0.\S2A=4.883892\RMSD=0.000e+00\RMSF=1.171e-05\Dipole=3.3671633,2.524
0766,8.9138892\Quadrupole=-9.9892954,3.8371477,6.1521476,-13.0542524,-
20.853663,-19.9731126\PG=C01 [X(C7H7Fe1N3O5S3)]\\@
```

Conformation c2

```
1\1\GINC-NODE167\FOpt\UB3LYP\6-31+G(d,p)\C7H7Fe1N3O5S3\SDS\05-Apr-2021
\0\\#N UB3LYP/6-31+G(d,p) Int(Grid=UltraFine) SCF(QC,Tight) SCRF=(Read
,Solvent=Water,SMD) Opt=(Redun) Freq(NoRaman) Symmetry(None) test\\[Fe
(NO)2(TBA)(SSO3)]- conf2 SSO3g- g+ tr g- opt water\\-1,2\Fe,1.26625245
74,1.2652677199,0.1338224324\S,-1.0897256192,1.2592421934,-0.398455701
6\S,2.4625618427,-0.0145882779,-1.3939290139\S,2.8132427239,-1.8629602
73,-0.4071625096\O,1.9649166308,4.0620622196,-0.3254224428\O,1.7585124
403,0.4763757323,2.8931389724\O,3.3471481957,-2.7707473478,-1.47563496
24\O,1.4947222425,-2.353629318,0.134315077\O,3.807098018,-1.6256561696
,0.6907900743\N,1.6264508333,2.9326415046,-0.2448702715\N,1.4984880668
,0.6896055627,1.7603562751\N,-1.1759212375,-1.3260518673,0.3075763142\
H,-1.6812140409,-2.1633503736,0.5808004681\H,-0.1559105927,-1.40946981
49,0.2488496908\C,-1.8437113561,-0.2326490809,0.000380926\C,-3.3239123
633,-0.3152652789,-0.0325399747\C,-4.1123256043,0.7621899295,0.4092249
229\H,-3.6365013953,1.6604202694,0.7868296875\C,-5.5032605777,0.666898
304,0.3886472418\H,-6.1029065265,1.4977050612,0.7472854136\C,-6.122411
0807,-0.4927520477,-0.0895508474\H,-7.2059256564,-0.5607165075,-0.1119
268827\C,-5.344611862,-1.563108618,-0.542483184\H,-5.8198832518,-2.459
9736498,-0.9273866329\C,-3.9526357023,-1.4815789701,-0.5092869279\H,-3
.3597474099,-2.3102417765,-0.8841289205\\Version=EM64L-G09RevB.01\HF=-
3269.7233888\S2=2.487335\S2-1=0.\S2A=4.877468\RMSD=0.000e+00\RMSF=2.39
1e-05\Dipole=-9.776711,2.5432834,1.1331895\Quadrupole=-5.0904194,-1.17
2563,6.2629823,30.8230228,3.3845788,-2.05906\PG=Unknown\\@
```

Conformation c3

```
1\1\GINC-NODE168\FOpt\UB3LYP\6-31+G(d,p)\C7H7Fe1N3O5S3\SDS\05-Apr-2021
\0\\#N UB3LYP/6-31+G(d,p) Int(Grid=UltraFine) SCF(QC,Tight) SCRF=(Read
,Solvent=Water,SMD) Opt=(Redun) Freq(NoRaman) Symmetry(None) test\\[Fe
(NO)2(TBA)(SSO3)]- conf3 SSO3g- g+ g+ g+ opt water\\-1,2\Fe,1.29909512
82,1.0748642688,0.3233578361\S,-0.9458717934,0.903038007,-0.5694084998
\S,2.7280312106,0.4732818089,-1.4120674921\S,2.8796760099,-1.647081747
6,-1.3157520166\O,1.9016332423,3.8844361727,0.846091563\O,1.7555949528
,-0.70655771,2.5819661269\O,3.6301883602,-2.0197350522,-2.5634476225\O
,1.4893963073,-2.2153132469,-1.3015855546\O,3.6428784987,-2.0026060891
,-0.0717777641\N,1.5882720333,2.7893049731,0.5238186178\N,1.5091496871
,-0.037498364,1.6419612135\N,-2.3441195356,-1.3081208958,-0.7944275033
\H,-2.8853866296,-2.0995718791,-0.4604571077\H,-2.35588994,-1.11332822
35,-1.7902317999\C,-1.666002032,-0.5407132754,0.0387375878\C,-1.615372
0676,-0.9462446005,1.4586487365\C,-1.7037033447,0.0206763777,2.4761388
09\H,-1.8035518406,1.0687338166,2.2161486067\C,-1.6878154189,-0.369773
0462,3.8137722554\H,-1.7721024807,0.3811434119,4.5931146497\C,-1.56053
52107,-1.7219103392,4.1504724397\H,-1.5365031875,-2.021618772,5.193898
2072\C,-1.4590368189,-2.6869300742,3.1435208641\H,-1.3455692864,-3.735
4399362,3.4006100038\C,-1.4945711052,-2.3060510626,1.8021088075\H,-1.3
934997936,-3.0591000884,1.026855238\\Version=EM64L-G09RevB.01\HF=-3269
.7167708\S2=2.501335\S2-1=0.\S2A=4.931737\RMSD=0.000e+00\RMSF=9.249e-0
6\Dipole=-9.0351314,1.3796869,4.6420482\Quadrupole=-21.4640099,5.81001
67,15.6539932,32.3507692,18.2281757,-11.7467371\PG=Unknown\\@
```

Conformation c6

```
1\1\GINC-NODE153\FOpt\UB3LYP\6-31+G(d,p)\C7H7Fe1N3O5S3\SDS\05-Apr-2021
\0\\#N UB3LYP/6-31+G(d,p) Int(Grid=UltraFine) SCF(QC,Tight) SCRF=(Read
,Solvent=Water,SMD) Opt=(Redun) Freq(NoRaman) Symmetry(None) test\\[Fe
(NO)2(TBA)(SSO3)]- conf6 SSO3g- g+ g- g- opt water\\-1,2\Fe,1.21632051
12,1.1723549003,0.2119283221\S,-1.1408897161,1.4679033269,-0.302942737
4\S,2.5632786505,0.4758063694,-1.5551758891\S,3.409081012,-1.396361022
8,-0.9952065457\O,2.0828982437,3.9142409433,0.727369746\O,1.6830738237
,-0.5050892039,2.5424191193\O,4.1032153427,-1.8693169158,-2.241927562\
O,2.3065082655,-2.3235149716,-0.5766946288\O,4.3771613054,-1.136950144
9,0.1245876469\N,1.6428183718,2.8565393008,0.4328226158\N,1.4043571347
,0.0734235368,1.5507051485\N,-3.0535068873,-0.081364868,0.6132005532\H
,-3.6408006916,-0.9091787213,0.646993132\H,-3.3491179152,0.7301684539,
1.1459194091\C,-1.9612263571,-0.043565839,-0.1259764833\C,-1.551318301
2,-1.2866297843,-0.8058246465\C,-1.0267497091,-1.2396881673,-2.1096491
408\H,-0.8923260628,-0.2831084399,-2.6013937462\C,-0.7115261449,-2.421
6446085,-2.7776493666\H,-0.3239477468,-2.3784636048,-3.7906284057\C,-0
.8878300098,-3.6560535052,-2.1442982578\H,-0.6294108602,-4.5741051872,
-2.6634213316\C,-1.3882769197,-3.7085039116,-0.8388473938\H,-1.5100964
143,-4.6638974198,-0.3381891561\C,-1.7306867897,-2.532672314,-0.173323
5318\H,-2.1046773013,-2.5807025536,0.8448070644\\Version=EM64L-G09RevB
.01\HF=-3269.713024\S2=2.52583\S2-1=0.\S2A=5.044211\RMSD=0.000e+00\RMS
F=1.407e-05\Dipole=-10.768459,0.977414,1.6568161\Quadrupole=-27.833768
6,11.9153222,15.9184464,27.7389038,9.6242879,-4.2321823\PG=Unknown\\@
```

Conformation c7


```
1\1\GINC-NODE153\FOpt\UB3LYP\6-31+G(d,p)\C7H7Fe1N3O5S3\SDS\02-May-2021
\0\\#N UB3LYP/6-31+G(d,p) Int(Grid=UltraFine) SCF(QC,Tight) SCRF=(Read
,Solvent=Water,SMD) Opt=(Redun) Freq(NoRaman) Symmetry(None) test\\[Fe
(NO)2(TBA)(SSO3)]- conf7 SSO3g- g- tr g- opt water\\-1,2\Fe,1.20639291
57,1.2622654757,-0.0878890271\S,-1.2188969345,1.3789852559,-0.16742879
42\S,2.0829557983,-0.0826214102,-1.7597532099\S,1.847349935,-2.0896236
098,-1.0794485184\O,2.5612877419,3.8341083313,-0.2792346743\O,1.669634
3656,0.1384468472,2.5592012196\O,2.2647827471,-2.9246148469,-2.2563372
148\O,0.4044755585,-2.2979371833,-0.7212175581\O,2.7544865182,-2.28262
84202,0.1013275904\N,1.9334366464,2.8326361827,-0.3349854415\N,1.39336
73013,0.5182835823,1.4768313675\N,-1.0232166945,2.6719378959,-2.505701
2817\H,-1.4016008869,3.0356034965,-3.3743355332\H,-0.0246759509,2.7648
093785,-2.3620966515\C,-1.8067789984,2.1018009995,-1.6075839867\C,-3.2
667439128,2.1158375996,-1.860625766\C,-4.0713710179,1.03143245,-1.4662
983472\H,-3.6199767618,0.169978442,-0.9869255931\C,-5.4430760498,1.049
8905134,-1.7142105383\H,-6.0523571497,0.2016578991,-1.4182473953\C,-6.
0301710775,2.1529228259,-2.3430267697\H,-7.0999456941,2.1675926233,-2.
5284966978\C,-5.2382858042,3.2392329114,-2.7279712334\H,-5.6902116011,
4.1040284664,-3.2037408343\C,-3.8634145812,3.222554034,-2.4951787909\H
,-3.266754102,4.0848386218,-2.7756232832\\Version=EM64L-G09RevB.01\HF=
-3269.7194125\S2=2.482208\S2-1=0.\S2A=4.855778\RMSD=0.000e+00\RMSF=3.9
80e-05\Dipole=-7.6825445,9.4246155,-2.2165916\Quadrupole=1.9873818,-8.
5912002,6.6038184,7.9743735,21.9986486,-31.1535752\PG=Unknown\\@
```


Conformation c8


```
1\1\GINC-NODE164\FOpt\UB3LYP\6-31+G(d,p)\C7H7Fe1N3O5S3\SDS\01-May-2021
\0\\#N UB3LYP/6-31+G(d,p) Int(Grid=UltraFine) SCF(QC,Tight) SCRF=(Read
,Solvent=Water,SMD) Opt=(Redun) Freq(NoRaman) Symmetry(None) test\\[Fe
(NO)2(TBA)(SSO3)]- conf8 SSO3g- g- tr g+ opt water\\-1,2\Fe,1.06059807
43,1.4745761686,0.1690359647\S,-1.3462115039,1.4778927779,-0.152001526
4\S,2.073115367,-0.1392468948,-1.1630673545\S,1.7619476516,-2.02654363
71,-0.2077537047\O,2.1736983429,4.0147519694,-0.7444132052\O,1.3425124
211,1.1007852987,3.0476105565\O,2.2971766522,-3.0214317446,-1.19536539
27\O,0.2909046874,-2.1928486018,0.0304821199\O,2.5462555758,-2.0130729
541,1.0702798078\N,1.6815332131,2.9747226957,-0.4751999818\N,1.1582061
027,1.1543792507,1.8823874159\N,-0.8413835743,0.8967145918,-2.70946520
89\H,-1.0851852946,0.8330566778,-3.6927139073\H,0.0913500907,0.5908037
454,-2.4281378676\C,-1.7301527217,1.2870051963,-1.8150967772\C,-3.1183
937892,1.5275816562,-2.2698131351\C,-3.893610891,2.548612793,-1.690967
8043\H,-3.4687094791,3.1743117339,-0.9139253767\C,-5.1962715557,2.7751
039911,-2.1328904774\H,-5.7814752349,3.574852669,-1.6898002466\C,-5.74
54212396,1.9776032224,-3.1424737351\H,-6.7628941735,2.151370588,-3.479
586116\C,-4.9845206317,0.9529212703,-3.7143738702\H,-5.4109848481,0.32
20764415,-4.4880321748\C,-3.6761292027,0.7291759937,-3.2870968488\H,-3
.1058422815,-0.0862277379,-3.7206963196\\Version=EM64L-G09RevB.01\HF=-
3269.7201878\S2=2.495492\S2-1=0.\S2A=4.911592\RMSD=0.000e+00\RMSF=1.26
8e-05\Dipole=-7.073588,6.9848701,-4.5009554\Quadrupole=2.5175773,-21.2
091515,18.6915743,8.7650873,16.2653273,-5.1810931\PG=Unknown\\@
```


Conformation c9


```
1\1\GINC-NODE166\FOpt\UB3LYP\6-31+G(d,p)\C7H7Fe1N3O5S3\SDS\01-May-2021
\0\\#N UB3LYP/6-31+G(d,p) Int(Grid=UltraFine) SCF(QC,Tight) SCRF=(Read
,Solvent=Water,SMD) Opt=(Redun) Freq(NoRaman) Symmetry(None) test\\[Fe
(NO)2(TBA)(SSO3)]- conf9 SSO3g- g- g- g- opt water\\-1,2\Fe,0.95392159
13,1.4169424692,0.07598464\S,-1.4451258566,1.3613071802,-0.3076418905\
S,1.9369182058,-0.3679946229,-1.0363833383\S,1.7240347036,-2.065620510
6,0.2297489418\O,2.4159351846,3.7421288303,-0.9033634542\O,1.214510081
4,1.1824993188,2.9679858949\O,2.2594811028,-3.2039199976,-0.5928221549
\O,0.2671175372,-2.2388399563,0.551500554\O,2.5454972202,-1.8263640302
,1.4643966263\N,1.7535441187,2.8060293454,-0.6150362124\N,1.0016179085
,1.1939524686,1.8060183814\N,-2.700651702,0.7116634356,-2.5170145595\H
,-2.9979325703,0.8254661906,-3.4811652869\H,-3.172084908,0.0102165964,
-1.9553412713\C,-1.7551365538,1.4741526676,-1.9990812407\C,-1.05567038
94,2.4018561669,-2.9112200815\C,-0.7381336555,3.7054944,-2.4917851309\
H,-0.9941859723,4.0257475706,-1.4879045685\C,-0.1206312159,4.591252915
7,-3.37305886\H,0.1067071831,5.6013824439,-3.0471335618\C,0.2062263907
,4.1800295819,-4.6699137311\H,0.6980854662,4.868963358,-5.3500321992\C
,-0.0963425947,2.8804488928,-5.0891138122\H,0.1664798761,2.5539966371,
-6.0904790933\C,-0.7343040573,1.9952351054,-4.2203794283\H,-0.95230088
43,0.9832934328,-4.5477743508\\Version=EM64L-G09RevB.01\HF=-3269.71591
29\S2=2.504902\S2-1=0.\S2A=4.953308\RMSD=0.000e+00\RMSF=5.378e-06\Dipo
le=-5.9758185,8.4041534,-6.4313713\Quadrupole=-8.0390899,-15.5155152,2
3.5546051,16.980333,9.8223497,-9.006366\PG=Unknown\\@
```


Conformation c12

1\1\GINC-NODE167\FOpt\UB3LYP\6-31+G(d,p)\C7H7Fe1N3O5S3\SDS\01-May-2021
\0\\#N UB3LYP/6-31+G(d,p) Int(Grid=UltraFine) SCF(QC,Tight) SCRF=(Read
,Solvent=Water,SMD) Opt=(Redun) Freq(NoRaman) Symmetry(None) test\\[Fe
(NO)2(TBA)(SSO3)]- conf12 SSO3g- g- g+ g+ opt water\\-1,2\Fe,1.1395654
531,1.0238475967,-0.7484291443\S,-1.281579919,1.2481762878,-0.63519024
99\S,1.8541538318,0.6882158914,-2.9327088503\S,1.64695387,-1.398687638
5,-3.2956696358\O,2.4087605182,3.5037984228,0.1257849799\O,1.736828921
7,-1.3376306783,0.8599953985\O,1.9501578793,-1.5490582384,-4.759927341
4\O,0.2346938872,-1.7924852534,-2.967981499\O,2.6444378223,-2.12251684
42,-2.4371333986\N,1.8448156968,2.5432097783,-0.2680235793\N,1.3979832
073,-0.4406149915,0.1687934892\N,-2.887067051,3.1963264475,0.072274589
9\H,-3.2495042295,4.1445379983,0.0683752401\H,-3.4392396142,2.47912745
05,0.5309307689\C,-1.7480905204,2.900698552,-0.529648876\C,-0.96249237
71,4.0231634274,-1.0833822842\C,-0.3584415611,3.915124865,-2.347981784
1\H,-0.4734218701,3.0047216746,-2.9257453151\C,0.3671354447,4.98570957
73,-2.8678233939\H,0.8168935465,4.9007221857,-3.8521337609\C,0.5183835
899,6.160570556,-2.1234528151\H,1.0966057703,6.9870723417,-2.525577835
6\C,-0.0731098609,6.2699750377,-0.8609420049\H,0.0508665987,7.17602290
2,-0.2760015352\C,-0.8215045063,5.2121957752,-0.3439617827\H,-1.263357
3113,5.2965467283,0.6440450969\\Version=EM64L-G09RevB.01\HF=-3269.7161
608\S2=2.514808\S2-1=0.\S2A=4.991681\RMSD=0.000e+00\RMSF=1.544e-05\Dip
ole=-5.7637923,11.1147784,5.5726954\Quadrupole=6.8968857,36.4737619,-4
3.3706476,-3.6138783,22.0154661,-27.3750618\PG=Unknown\\@

Conformation c15

1\1\GINC-NODE167\FOpt\UB3LYP\6-31+G(d,p)\C7H7Fe1N3O5S3\SDS\17-Apr-2021
\0\\#N UB3LYP/6-31+G(d,p) Int(Grid=UltraFine) SCF(QC,Tight) SCRF=(Read
,Solvent=Water,SMD) Opt=(Redun) Freq(NoRaman) Symmetry(None) test\\[Fe
(NO)2(TBA)(SSO3)]- conf15 SSO3tr g+ g- g- opt water\\-1,2\Fe,1.5085008
192,1.22226778,0.3458515537\S,-0.6861329831,1.4949021018,-0.699941372\
S,3.0159313545,2.3485259088,-1.0054251436\S,3.7507184464,0.9431104461,
-2.4269692745\O,1.560036018,2.7987180317,2.8023222396\O,2.3902971689,-
1.4857370115,0.9237267255\O,4.6674389015,1.7532042834,-3.2994137649\O,
2.5807341334,0.395891887,-3.1922587902\O,4.4853246959,-0.1317897732,-1
.6787965478\N,1.439674503,2.2627805709,1.7540255271\N,1.9615141273,-0.
4424885505,0.5747050328\N,-2.9786876107,0.2397100175,-0.5381341402\H,-
3.6071349267,-0.5538777014,-0.6127602075\H,-3.390477889,1.1604513167,-
0.4280168122\C,-1.6680434708,0.0841884748,-0.6235253934\C,-1.156112405
8,-1.3005907184,-0.6870065753\C,-0.1138634912,-1.6329003873,-1.5693518
181\H,0.3335417158,-0.8658290008,-2.1910114114\C,0.3271981772,-2.95212
93638,-1.6622775213\H,1.1222677873,-3.2033740795,-2.357300483\C,-0.249
0141294,-3.9451598606,-0.8631528972\H,0.1038412883,-4.9698722275,-0.93
12884624\C,-1.2772481694,-3.6175457248,0.0268067898\H,-1.7188636889,-4
.3828548049,0.6574751439\C,-1.7387618526,-2.3039993683,0.1106516417\H,
-2.5279045992,-2.0541193912,0.8131717429\\Version=EM64L-G09RevB.01\HF=
-3269.7148381\S2=2.515897\S2-1=0.\S2A=5.000913\RMSD=0.000e+00\RMSF=1.3
89e-05\Dipole=-10.7439356,-3.6566014,4.4238633\Quadrupole=-25.1511831,
29.5627735,-4.4115904,-16.9376199,43.613713,11.60728\PG=Unknown\\@

Conformation c16

1\1\GINC-NODE160\FOpt\UB3LYP\6-31+G(d,p)\C7H7Fe1N3O5S3\SDS\18-Apr-2021
\0\\#N UB3LYP/6-31+G(d,p) Int(Grid=UltraFine) SCF(QC,Tight) SCRF=(Read
,Solvent=Water,SMD) Opt=(Redun) Freq(NoRaman) Symmetry(None) test\\[Fe
(NO)2(TBA)(SSO3)]- conf16 SSO3g- tr tr g+ opt water\\-1,2\Fe,1.4515039
226,1.527903236,0.2671864187\S,-0.9677587388,1.4715480459,-0.108332647
3\S,2.1437845062,0.0006292779,-1.3201709603\S,2.1530203195,-1.90160024
97,-0.3537864449\O,2.6353298015,4.1021807145,-0.436231559\O,2.21681304
14,0.7507170864,2.9690670927\O,2.4760373214,-2.8668699692,-1.458374220
6\O,0.7885385797,-2.1389077101,0.2249390678\O,3.2172050399,-1.87435118
37,0.7044927043\N,2.0661662096,3.0804618028,-0.2569636593\N,1.80491024
32,0.9895339083,1.8874820702\N,-1.1526653238,3.2976769851,1.8318923189
\H,-1.662897182,3.8569263583,2.5071237297\H,-0.1401661015,3.3536812569
,1.8460163538\C,-1.7871410987,2.5051324243,0.9841546801\C,-3.268252628
8,2.547300485,0.9777793008\C,-4.0149487781,1.3853096345,0.7114156458\H
,-3.5053869637,0.4466936043,0.5238545614\C,-5.4082559697,1.431092632,0
.7141535696\H,-5.9744614732,0.5252629581,0.5211670254\C,-6.0726049369,
2.6362073084,0.9653842712\H,-7.1578826953,2.6704518512,0.9589686514\C,
-5.3368619036,3.7977111872,1.2208230446\H,-5.846737682,4.7384362154,1.
4038492808\C,-3.9428386955,3.7566184722,1.2341272675\H,-3.3856381784,4
.6712772631,1.4101824108\\Version=EM64L-G09RevB.01\HF=-3269.7198733\S2
=2.503561\S2-1=0.\S2A=4.95578\RMSD=0.000e+00\RMSF=1.301e-05\Dipole=-8.
3808881,10.1342019,3.5682355\Quadrupole=-3.9837222,3.7788541,0.2048681
,4.3929651,-4.7355146,13.0214765\PG=Unknown\\@

Conformation c17

1\1\GINC-NODE173\FOpt\UB3LYP\6-31+G(d,p)\C7H7Fe1N3O5S3\SDS\17-Apr-2021
\0\\#N UB3LYP/6-31+G(d,p) Int(Grid=UltraFine) SCF(QC,Tight) SCRF=(Read
,Solvent=Water,SMD) Opt=(Redun) Freq(NoRaman) Symmetry(None) test\\[Fe

```
(NO)2(TBA)(SSO3)]- conf17 SSO3g- tr tr g- opt water\\-1,2\Fe,1.4113054
343,1.4890028341,0.3233407294\S,-1.0111150657,1.2909422469,0.078803607
\S,2.0949560975,0.0558220483,-1.352386771\S,2.3306324175,-1.8559760776
,-0.4343586626\O,2.3652478146,4.1567218694,-0.3886772498\O,2.336324469
,0.584187776,2.9366823556\O,2.5864122555,-2.7843686302,-1.5870629379\O
,1.0590848613,-2.1898071214,0.2898280733\O,3.5047573288,-1.7706411156,
0.4971582347\N,1.8886588882,3.0934295544,-0.1829082991\N,1.8721078855,
0.8797028055,1.8909133376\N,-1.19403442,3.1947800244,1.9435636666\H,-1
.696038195,3.8901313482,2.4854390141\H,-0.1907901675,3.1318484259,2.07
49321505\C,-1.8251728912,2.4170005809,1.0800034597\C,-3.3013099625,2.5
248360162,1.0109310197\C,-3.9737368778,2.3729510772,-0.2149895029\H,-3
.4090475164,2.1866242334,-1.1218050241\C,-5.3619282165,2.4890882428,-0
.2713818741\H,-5.8701052179,2.3856573347,-1.2250142714\C,-6.0957982403
,2.7391311191,0.8930440249\H,-7.1775500287,2.8212264223,0.8470917103\C
,-5.4345538872,2.8798351795,2.1172274271\H,-5.9995135462,3.0608140126,
3.0263290093\C,-4.0447907928,2.7806516495,2.1789616704\H,-3.5461134827
,2.8684205385,3.1389560454\\Version=EM64L-G09RevB.01\HF=-3269.7198363\
S2=2.504687\S2-1=0.\S2A=4.958886\RMSD=0.000e+00\RMSF=1.363e-05\Dipole=
-8.841769,10.014727,3.7987479\Quadrupole=-6.752355,-2.1498313,8.902186
3,4.610269,-3.3667197,12.1698829\PG=Unknown\\@
```

Conformation c18

```
1\1\GINC-NODE169\FOpt\UB3LYP\6-31+G(d,p)\C7H7Fe1N3O5S3\SDS\20-Apr-2021
\0\\#N UB3LYP/6-31+G(d,p) Int(Grid=UltraFine) SCF(QC,Tight) SCRF=(Read
,Solvent=Water,SMD) Opt=(Redun) Freq(NoRaman) Symmetry(None) test\\[Fe
(NO)2(TBA)(SSO3)]- conf18 SSO3g- tr g+ g+ opt water\\-1,2\Fe,1.0583113
818,1.5484244954,-0.6944863173\S,-1.3669866728,1.2882866482,-0.5855791
378\S,1.7778793034,-0.6311686995,-1.0120843761\S,2.0605672009,-1.45008
20285,0.9345678815\O,1.8973297662,2.9627194212,-3.110975219\O,2.129115
7128,2.6682111517,1.7738698716\O,2.3834695007,-2.8950478826,0.67968346
39\O,0.783922076,-1.2841911588,1.7075230344\O,3.2046096428,-0.72006837
43,1.5777127736\N,1.4586409368,2.3434309712,-2.2020846227\N,1.61978125
5,2.1746088313,0.8295631706\N,-3.1877303745,2.0871654106,1.1228024691\
H,-3.6799192577,2.7587431811,1.7029780914\H,-3.6129160703,1.175921359,
0.9886995097\C,-2.0482808204,2.4022731588,0.528338638\C,-1.4534537184,
3.71564726,0.8562687302\C,-0.8643456314,4.5043038936,-0.1477563532\H,-
0.8380554522,4.1454501632,-1.1706723232\C,-0.345522651,5.7598487233,0.
1649312253\H,0.0902603062,6.3706529945,-0.6195602202\C,-0.3853886738,6
.2295502516,1.4822961739\H,0.028191062,7.2040386041,1.7239903478\C,-0.
9555332565,5.4427420758,2.4879961064\H,-0.9769247566,5.7975746337,3.51
36681102\C,-1.4957724779,4.1943260902,2.1791876131\H,-1.922779331,3.58
31368252,2.9683623587\\Version=EM64L-G09RevB.01\HF=-3269.7168319\S2=2.
525412\S2-1=0.\S2A=5.045272\RMSD=0.000e+00\RMSF=5.801e-05\Dipole=-7.57
98944,9.9232012,-0.1503579\Quadrupole=-8.9559024,12.1089359,-3.1530334
,2.3439629,-20.231692,18.6030556\PG=Unknown\\@
```

Conformation c21

```
1\1\GINC-NODE169\FOpt\UB3LYP\6-31+G(d,p)\C7H7Fe1N3O5S3\SDS\19-Apr-2021
\0\\#N UB3LYP/6-31+G(d,p) Int(Grid=UltraFine) SCF(QC,Tight) SCRF=(Read
,Solvent=Water,SMD) Opt=(Redun) Freq(NoRaman) Symmetry(None) test\\[Fe
(NO)2(TBA)(SSO3)]- conf21 SSO3g- tr g- g- opt water\\-1,2\Fe,1.2720570
252,0.9739215852,0.9064857726\S,-1.1036531185,0.6705940201,0.412552602
5\S,2.2763447418,0.7730791578,-1.1727720868\S,2.6655311536,-1.30275369
09,-1.4494578188\O,1.7523794668,3.6956026381,1.8466943509\O,2.21476183
94,-1.1186574592,2.7125341966\O,3.1352536712,-1.4005277648,-2.87315457
61\O,1.3830784799,-2.05124486,-1.2246811376\O,3.7319489089,-1.70912803
83,-0.4733923279\N,1.4928008589,2.6414911363,1.3781603332\N,1.76937470
01,-0.3385049483,1.9436763706\N,-3.2064872979,2.1850598548,0.827873511
8\H,-3.8372803711,2.7585572348,1.3783926846\H,-3.5111402582,1.90213409
18,-0.0975369876\C,-2.0418280891,1.7894309035,1.3157324415\C,-1.640316
4538,2.3315834445,2.6317312891\C,-1.0294365949,1.5029573106,3.58927169
72\H,-0.8486947279,0.4581131611,3.3624899738\C,-0.6856854545,2.0161730
345,4.8390448452\H,-0.2311181259,1.3656108948,5.579644282\C,-0.9234496
843,3.362593952,5.1365288198\H,-0.6440137375,3.7619806381,6.1068482851
\C,-1.5182513307,4.1947662155,4.1829768431\H,-1.6930032971,5.242835948
,4.4053383915\C,-1.8850700672,3.6832692705,2.9379631839\H,-2.331405613
1,4.3387167852,2.1965108938\\Version=EM64L-G09RevB.01\HF=-3269.7167329
\S2=2.528077\S2-1=0.\S2A=5.059398\RMSD=0.000e+00\RMSF=1.826e-05\Dipole
=-9.1339541,8.0247047,7.5335126\Quadrupole=-15.5419945,9.2419904,6.300
0042,-0.2824781,12.5413086,2.5888537\PG=Unknown\\@
```
